# Marine *Pseudovibrio* sp. as a Novel Source of Antimicrobials

**DOI:** 10.3390/md12125916

**Published:** 2014-12-09

**Authors:** Susan P. Crowley, Fergal O’Gara, Orla O’Sullivan, Paul D. Cotter, Alan D. W. Dobson

**Affiliations:** 1 Teagasc, Moorepark Food Research Centre, Fermoy Co. Cork, Ireland; E-Mails: susan.crowley@teagasc.ie (S.P.C.); orla.osullivan@teagasc.ie (O.O.S.); 2 School of Microbiology, University College Cork, Western Road, Cork, Ireland; E-Mails: f.ogara@ucc.ie (F.O.G.); a.dobson@ucc.ie (A.D.W.D.); 3 Biomerit Research Centre, University College Cork, Western Road, Cork, Ireland; 4 Alimentary Pharmabiotic Centre, Biosciences Institute, University College Cork, Western Road, Cork, Ireland; 5 Marine Biotechnology Centre, Environmental Research Institute, Lee Road, Cork, Ireland

**Keywords:** antimicrobial, bioactives, marine, microorganism, resistance

## Abstract

Antibiotic resistance among pathogenic microorganisms is becoming ever more common. Unfortunately, the development of new antibiotics which may combat resistance has decreased. Recently, however the oceans and the marine animals that reside there have received increased attention as a potential source for natural product discovery. Many marine eukaryotes interact and form close associations with microorganisms that inhabit their surfaces, many of which can inhibit the attachment, growth or survival of competitor species. It is the bioactive compounds responsible for the inhibition that is of interest to researchers on the hunt for novel bioactives. The genus *Pseudovibrio* has been repeatedly identified from the bacterial communities isolated from marine surfaces. In addition, antimicrobial activity assays have demonstrated significant antimicrobial producing capabilities throughout the genus. This review will describe the potency, spectrum and possible novelty of the compounds produced by these bacteria, while highlighting the capacity for this genus to produce natural antimicrobial compounds which could be employed to control undesirable bacteria in the healthcare and food production sectors.

## 1. Introduction

Antibiotic resistance among pathogenic microorganisms is becoming ever more common. Recent data published by the Centre for Disease Control and Prevention (CDC), estimated that the number of illnesses and deaths caused by antibiotic resistance exceeded 2 million and 23,000, respectively, in the United States [1] and 25,000 deaths annually in Europe are attributed to resistant hospital infections [2]. Drug resistant pathogens are currently placing a heavy financial burden on health systems worldwide with infections due to selected multidrug-resistant bacteria in the EU estimated to result in extra health-care costs and productivity losses of at least €1.5 billion each year [3]. These worrying trends of increased multidrug resistance, has led to a call by The Infectious Disease Society of America, for the delivery of ten new antibiotic drugs by 2020 [4]. Unfortunately, however, the number of new antibiotics being developed has decreased, rather than increased, in recent years [5]. The ever reducing rate at which novel and potent antimicrobials are emerging [6] means we are becoming ever more dependent on our current arsenal of antibiotics, which are rapidly losing potency. There is an urgent need for the discovery and supply of novel and potent antimicrobials from natural resources. Nearly 10% of known biologically active natural products are of microbial origin [6], with terrestrial bacteria considered well studied when compared to marine bacteria, which have in recent years been increasingly recognised for their biotechnological potential [7]. Current estimates for the global market for Marine Biotechnology products and processes stand at €2.8 billion (2010) [8]. Not surprisingly, focus has recently shifted to the oceans as a potential source for natural product discovery. Antimicrobials derived from marine microorganisms are of particular interest due to the suite of compounds being produced [9,10,11,12,13,14]. The aim of this review is to highlight a marine-derived bacteria: *Pseudovibrio* spp., which have shown themselves to be producers of bioactives and propose that this genus is a novel and a relatively untapped source of antimicrobial compounds.

## 2. *Pseudovibrio* sp.

In recent years, interest in marine-derived *Pseudovibrio* has increased due to the dominance of the genus amongst host-associated microbial communities, the antimicrobial ability of the members and the potency, broad-spectrum and novelty of the compounds produced by these bacteria [15,16,17,18,19]. Members of this genus are of the Rhodobacteracea family, Rhodobacterales order and alphaproteobacteria class. They have been obtained from numerous marine sources such as the surfaces of tunicates [15], sea squirts [20], coral [21], seawater [22], algae [23] and from a great variety of sponges worldwide, such as *Axinella corrugata*, *Mycale laxissima* from Florida [24]; *E. discophorus* from Portugal [25]; *Irciniidae* sponges also from Portuguese waters [26]; *Didiscus oxeata* from Jamaica [27]; *P. magna*, *C. aurea*, *M. microsigmato* from Brazil [28]; *A. dissimilis*, *Polymastia boletiformis* and *H. simulans* from Ireland [12], *S. carnosus*, *Leucosolenia* sp. from Lough Hyne, Ireland and *Amphilectus fucorum*, *Eurypon major* also from Irish coastal waters [29].

### 2.1. Characterisation of the Genus Pseudovibrio

Even though the marine genus *Pseudovibrio* has been isolated throughout the marine environment, it was described for the first time only ten years ago. To date, four species have been described, *i.e.*, *P. denitrificans*, *P. ascidiaceicola*, *P. japonicas* and *P. axinellae* sp. nov. The first strain of the genus *Pseudovibrio* to be identified, a *P. denitrificans* isolate, was sourced from coastal seawater in 2004 [22]. More specifically, Shieh and colleagues isolated two denitrifying strains designated DN34T and DN33, from sea-water samples collected in Nanwan Bay, Kenting National Park, Taiwan which were named *Pseudovibrio* gen. nov. Characteristics associated with this species can be seen in Table 1 [22]. In 2006, based on the results of phylogenetic and phenotypic analyses of two strains isolated from ascidians (sea squirts), a novel species, *P. ascidiaceicola* sp. nov. was proposed [20]. In addition to the results in Table 1, tests for β-glucosidase, arginine dihydrolase and urease are positive. Indole is produced from tryptophan and gelatin and aesculin is hydrolysed [20]. *Pseudovibrio japonicas* strain WSF2T was isolated in 2007, from surface seawater off the coastline of the Boso Peninsula, Japan, and examined [30]. Tests in addition to those outlined in Table 1 also yielded positive results for alkaline phosphatase, esterase (C4), esterase lipase (C8), leucine arylamidase, valine arylamidase, trypsin, acid phosphatase, naphthol-AS-BI-phosphohydrolase and β-galactosidase [30]. More recently in 2013, O’ Halloran *et al.* [31], described strain Ad2T which, on the basis of phylogenetic analysis, DNA-DNA hybridization and differential phenotypic characteristics, was proposed as the type strain of a novel species, for which the name *Pseudovibrio axinellae* sp. nov. was proposed. Tests in addition to those mentioned in Table 1 revealed that aesculin, casein, DNA and gelatin are hydrolysed, while starch is not [31].

**Table 1:** General characteristics of *P. denitrificans*, *P. ascidiaceicola*, *P. japonicas* and *P. axinellae* sp. nov.

Characteristics	*P. denitrificans*	*P. ascidiaceicola*	*P. japonicas*	*P. axinellae*
Gram reaction	Negative	Negative	Negative	Negative
Oxidase	Positive	Positive	Positive	Positive
Catalase	Positive	Positive	Positive	Positive
Cell shape	Straight/curved rod (exponential-early stationary phase)	Straight/curved rod (exponential-early stationary phase) Predominantly cocci (late stationary phase)	Rod shaped	Rod shaped
Aerobic/anaerobic	Facultatively anaerobic	Facultatively anaerobic	Facultatively anaerobic	Facultatively anaerobic
Motile	Lateral or subpolar flagella	Subpolar flagella	Lateral or subpolar flagella	Subpolar flagella
Temperature tolerances	Grow at 20 °C–35 °C but not at 4 °C or 45 °C	Grow at 10 °C–30 °C	Grow at 15 °C–30 °C but not at 8 °C or 35 °C	Grow at 10 °C–30 °C
Halophilic	Grow at 2%–6% NaCl	Grow at 3%–5% NaCl	Grow at 1%–6% NaCl	Grow at 2%–4% NaCl
Substrates utilized for growth	Galactose, glucose, mannose, sucrose and trehalose but not d-arabinose, cellobiose, dulcitol, glycerol, inositol, lactose, mannitol, sorbitol or xylose.	Dextrin, Tween 80, *N*-acetyl-d-galactosamine, *N*-acetyl-d-glucosamine, l-fucose, d-galactose, α-d-glucose, myo-inositol, maltose, d-mannose, d-meli-biose, d-raffinose, sucrose, d-trehalose, turanose, d-gluconic acid, β-hydroxybutyric acid, dl-lactic acid, succinic acid, l-alanine, l-alanyl glycine, l-glutamic acid, glycyl-l-aspartic acid, glycyl-l-glutamic acid, hydroxyl-l-proline, l-proline, l-serine, inosine, uridine, thymidine, 2-amino ethanol, glycerol, d-glucose 6-phosphate.	d-glucose, maltose, d-mannose and sucrose, d-fructose, d-galactose, α-lactose or d-xylose negative results for arginine dihydrolase, lysine decarboxylase, ornithine decarboxylase, lipase (C4), cysteine arylamidase, chymotrypsin, α-galactosidase, β-glucuronidase, α-glucosidase, β-glucosidase, *N*-acetyl-β-glucosamidase, α-mannosidase and α-fucosidase utilisation.	Dextrin, Tween 40, Tween 80, l-fucose, α-d-glucose, myo-inositol, maltose, d-mannose, raffinose, sucrose, trehalose, turanose, pyruvic acid methyl ester, d-gluconic acid, d-glucuronic acid, β-hydroxybutyric acid, α-ketobutyric acid, α-ketoglutaric acid, dl-lactic acid, succinic acid, bromosuccinic acid, d-alanine, l-alanine, l-alanyl glycine, l-aspar-agine, l-aspartic acid, l-glutamic acid, glycyl l-aspartic acid, glycyl l-glut-amic acid, hydroxyl-l-proline, l-proline, l-serine, inosine, uridine, thymidine, 2-aminoethanol, glycerol.

### 2.2. Bioactivity of the genus Pseudovibrio

The most commercially significant characteristic of *Pseudovibrio* strains is the production of secondary metabolites, which has been reported in many studies and will be discussed in detail below. Over the last decade there has been a considerable increase in the number of studies relating to the antimicrobial activity of a variety of bioactive compounds by *Pseudovibrio* spp. These studies highlight that this genus has the potential to be a particularly promising source of novel metabolites. Two families of enzymes, *i.e*., polyketide synthases (PKS) and non-ribosomal peptide synthetases (NRPS), and their hybrids (PKS/NRPS) are of particular importance in the production of various secondary metabolites, many of which are important/natural products, across a wide range of microorganisms. Analysis of the presence of *PKS* and *NRPS* genes is often employed as a means of determining the likelihood that a microorganism has the potential to produce new bioactive compounds. For example, of the four *Pseudovibrio* cultures by Kennedy *et al*. [32] from the marine sponge *H. simulans,* two of which had 99% 16S rRNA gene identity match to *P. ascidiaceicola* F10102., three were found to contain both putative *PKS* and *NRPS* genes, suggesting a high potential for secondary metabolite production. In 2009, this group assessed the bioactivity of these *Pseudovibrio* bacteria [19]. The three *Pseudovibrio* strains, PV1, PV2, PV4, that contained *PKS* and *NRPS* genes exhibited antimicrobial activity against methicillin-resistant *S. aureus*, *B. cereus*, *E. coli* and *B. subtilis*. Interestingly, *Pseudovibrio* strain PV3 which did not contain *PKS* and *NRPS* genes, did not show antimicrobial activity against the target strains [19].

The presence of *PKS* and *NRPS* genes that might be involved in the production of secondary metabolites by sponge-associated microorganisms was also investigated in a study by Graça *et al.* [25]. In agreement with previous studies [33], it was found that the majority of bioactive bacteria in which *PKS-I* and *NRPS* genes were detected were *Pseudovibrio*. More specifically, Graça *et al.* found that of the 212 bacteria isolated from the marine sponge *E. discophorus*, 31% produced antimicrobial compounds. Of these 66 bioactive-producing isolates, the most bioactive genus was *Pseudovibrio* (47%) with bioactivity observed against *B. subtilis* ATCC6633, *S. aureus* MRSA and *Aliivibrio fischeri* CECT 524 [25]. This high level of activity within the genus has also been reported by Flemer *et al.* 2011 [34]. In that case out of the thirty *Pseudovibrio* strains isolated, 27 (90%) exhibited antimicrobial activity against at least two of three clinically relevant bacteria strains tested *i.e.*, *E. coli* NCIMB 12210, *B. subtilis* IA40, and *S. aureus* NCIMB 9518 [34]. The bioactivity profiles observed indicated the production of different antimicrobial compounds and again highlighted the broad range of activities associated with strains from this genus. This team also studied bioactive bacteria from the marine sponges *A. fucorum* and *E. major* [29]. In this instance, from the 409 bacterial strains isolated from both sponges and tested for antifungal and antibacterial activity, all of the strains exhibiting antibacterial activity were *Pseudovibrio* spp. More specifically, eight out of twelve *Pseudovibrio* strains isolated demonstrated activity. This activity was observed against at least one of the three targets employed, *i.e*., the aforementioned *E. coli* NCIMB 12210 as well as *B. subtilis* IE32 and *S. aureus* NC000949 [29].

Particularly broad spectrum activity by *Pseudovibrio* strains was demonstrated by Santos *et al.* [28]. *Pseudovibrio* strain Pm31 isolated from the sponge *P. magna*, strain Ca31 from *C. aurea* and strain Mm37 from *Mycale microsimatosa* demonstrated stable antimicrobial activity against Gram-positive targets *Corynebacterium fimi* NCTC 7547, *Enterococcus faecium* ATCC 19434, *Enterococcus faecalis* V583 (vancomycin), *E. faecalis* 5AE (vancomycin), *S. aureus* ATCC 29213, *S. aureus* 42AE (MRSA), *S. aureus* 559a (community-associated MRSA), *Staphylococcus epidermidis* ATCC 12228, *S. epidermidis* 57s (ampicillin, ciprofloxacin, penicillin, tetracycline), *Staphylococcus haemolyticus* ATCC 2997, *S. haemolyticus* 109s (ampicillin, gentamicin, oxacillin, penicillin), *Staphylococcus hominis* ATCC 27844, and against Gram-negative *Acinetobacter calcoaceticus* 56AE, *E. coli* 54 AE (ampicillin, chloramphenicol, trimethoprim/sulfamethoxazole, tetracyline), *P. aeruginosa* ATCC 27853, *P. aeruginosa* 3AE (aztreonam, piperacillin/tazobactam), and *Salmonella enterica* 4AE. It was noted that although activity was seen against both Gram-positive and Gram-negative target strains, the *Pseudovibrio* strains were more effective against Gram-positive bacteria. Tests to characterize the bioactive compound produced by the *Pseudovibri*o strains showed that the substances were resistant to the action of all proteolytic enzymes tested suggesting that these antimicrobial substances are not ribosomal proteins. Biofilm production by the isolated strains was observed and was particularly apparent when strains Pm31 and Mm37 were studied [28]. It has been hypothesized that the ability to produce antibacterial compounds combined with an enhancement in bioﬁlm formation may give bacteria a selective advantage and possible dominance over other surface-attached bacteria [35].

O’Halloran *et al.* [33], referred to briefly above, also demonstrated broad spectrum activity from among a population of 73 *Pseudovibrio* isolates from the marine sponges *A. dissimilis*, *P. boletiformis* and *H. simulans.* This involved an initial screen using deferred antagonism assays from which it was revealed that 62 isolates (84.9%) demonstrated antimicrobial activity against at least one of the indicator strains tested. The majority of the isolates showed activity against *E. coli* (58; 79.5%), *S. Typhimurium* (54; 74%), *B. subtilis* (49; 67.1%), *S. aureus* (54; 74.0%), MRSA (48; 65.8%), vancomycin intermediate *S. aureus* (VISA) (45; 61.6%), hVISA (47; 64.9%), *C. perfringens* (60; 82.2%) and *C. difficile* (55; 75.3%). Activity was also observed against *Yersinia enterocolitica* (1; 1.4%), *B. cereus* (4; 5.5%), *E. faecium* (6; 8.2%), vancomycin-resistant *Enterococcus* (VRE) (1; 1.4%) and *L. monocytogenes* (2; 2.7%). Fourteen different antimicrobial activity spectra were identified suggesting and that the *Pseudovibrio* spp. may be producing a number of different antimicrobial compounds. The *Pseudovibrio* isolates were also screened for the presence of *PKS* genes, using degenerate PCR, with keto synthase gene fragments being found in all 73 isolates.

**Figure 1:**
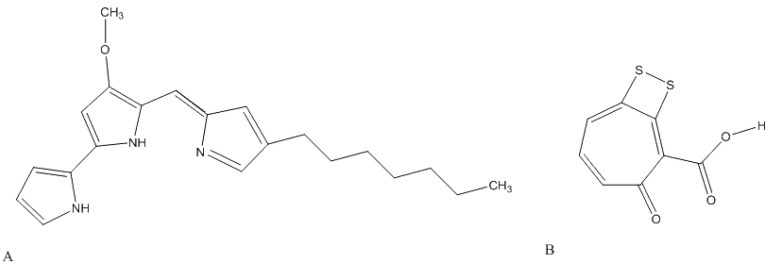
Structures of *Pseudovibrio*-related antimicrobial compounds [39]: (**A**) Heptylprodigiosin; (**B**) Tropodithietic acid.

Although secondary metabolite production and antimicrobial activity has been shown in many *Pseudovibrio*-related studies, in many cases the corresponding compounds have not been the focus of further analysis. However, some studies have characterised the relevant bioactive compounds. In one case, isolate Z143, a bacterium from a Philippine tunicate which had 100% 16S rRNA gene similarity with the *P. denitrificans* type strain DN34 was reported in 2007 by Sertan de-Guzman [15] as the first α-proteobacterium to produce the red pigment heptylprodigiosin (Figure 1A) also known as 16-methyl-15-heptyl-prodiginine, which shows anti-*Staphylococcus aureus* activity. Vizcaino *et al.* [21] revealed the production of a novel polypeptide, pseudovibrocin, which was isolated from three unique coral-derived bacteria with 99% 16S rRNA gene similarity to *P. denitrificans* that were capable of inhibiting Gram-positive and Gram-negative bacteria. An acetone extract of the associated cell-free supernatant was found to inhibit *Kocuria* *rhizophila* and a methanol extract inhibited *B. subtilis*, *Vibrio harveyi* and *V. coralliilyticus*. High-performance liquid chromatography analysis of the methanol extract suggested the presence of at least two antibiotics, one of which shown to be pseudovibrocin. Geng and Belas [16] studied the biosynthesis of tropodithietic acid (TDA), a tropolone antibiotic, by a number of strains. In addition to other members of the Roseobacteracea family, *i.e.*, *Silicibacter* sp. TM1040 and *Phaeobacter gallaeciensis* (both genera known to be TDA producers), *Pseudovibrio* sp. JE062, previously isolated by Enticknap *et al.* [24] was also found to be a TDA producer. The twelve genes required for TDA biosynthesis in strain JE062 were identified by transposon insertion mutagenesis and the organization of a number of the associated genes, *tdaA-F*, was found to be identical to that of the other bacteria in this study. The production of TDA by another *Pseudovibrio* sp., strain D323 (Figure 1B) has also been reported by Penesyan *et al.* [23]. This marine epiphytic strain was 99% 16S rRNA gene identical to *P. ascidiaceicola* (Genbank Acc. #AB175663) and exhibited antimicrobial activity against target strains from the phyla Alphaproteobacteria, Gammaproteobacteria, Bacteroidetes, Firmicutes and Actinobacteria. The authors in particular noted that TDA produced by D323 was highly active against *Nautella* sp. R11, which causes disease in a marine seaweed *Delisea pulchra*, thereby supporting the hypothesis that these host-associated bacteria serve as a defence against potential pathogenic surface colonisers.

Genome-based methods such as comparative bacterial genomics [36] and “genome scanning” of sequenced genomes of natural-product-producing bacteria [37] can also lead to valuable information on the diversity of bacterial species and can lead to bioactive product discovery. In this regard it is relevant that Bondarev *et al.* [38] carried out analysis of the genomes of two *Pseudovibrio* strains, JE062 and FO-BEG1, that originated from the coast of Florida. At the time of writing, strain FO-BEG1 is the only *Pseudovibrio* for which a fully sequenced closed genome sequence has been reported.

Genes involved in the biosynthesis of *TDA* were detected in FO-BEG1, and it was confirmed through culture-based methods that the strain is indeed a *TDA* producer. Although *TDA* production has been reported previously by other *Pseudovibrio* sp. [23,40], it is not clear how widely distributed this trait is. However, our own analysis of nine antimicrobial producing *Pseudovibrio* strains reveals the absence of *TDA* gene clusters from seven of these strains, indicating that this cluster may not be widely distributed [41]. Even in situations where the cluster is present, it is notable that culture conditions can impact on the production of *TDA* [42]. Indeed, *TDA* production has been detected during bacterial growth under static conditions [38], but not during incubation under agitation in broth, except for a brief period at approximately 10 hours after inoculation [16]. Another notable feature of the Bondarev study was the identification of genes predicted to encode a hybrid *NRPS-PKS* system in strain FO-BEG1, which resembled those previously associated with members of the Enterobacteriaceae [43]. More specifically, this cluster corresponds to a 50 kb genomic island which is architecturally similar to a *NRPS-PKS* system reported by Nougayrède *et al.* [44] and determined to be colibactin (an *E. coli* metabolite). Similarities in the architecture between these two *NRPS-PKS* systems led to the suggestion that the product of the *Pseudovibrio* FO-BEG1 cluster is colibactin. Again, it is not clear to what extent this cluster is distributed among *Pseudovibrio* sp. so we have employed a previously designed PCR primer set [44] to determine the distribution of the *NRPS-PKS* system within our nine *Pseudovibrio* strains, and detected the presence of these genes in all nine strains [41]. Romano *et al*. [45] also demonstrated the astonishing diversity of the exo-metabolome (extracellular metabolites) of strain FO-BEG1 and the drastic effect that phosphate limitation can have on its composition. More specifically, results showed that low phosphate concentrations can induce the production of secondary metabolites in *Pseudovibrio* FO-BEG1. Under phosphate limitation, a higher production of phenolic and polyphenolic compounds was also observed by Romano *et al.* [45] when cells entered stationary phase. It was suggested by the authors that some of these compounds may be tropone derivatives, of which TDA is an example, which are commonly produced by bacteria of the *Roseobacter* clade and can have antibacterial activity. Indeed members of the *Roseobacter* clade produce TDA in addition to an uncharacterised yellow pigment, which may be the same pigment produced in this study by *Pseudovibrio* under phosphate-limited conditions and entering stationary phase. In addition, several masses were predicted to correspond to cyclic dipeptides that resemble antimicrobials produced by *Roseobacter* strains isolated from marine sponges. Given the phylogenetic and physiological similarity between *Roseobacter* and *Pseudovibrio* bacteria, the authors reasoned that *Pseudovibrio* may also release such compounds into the medium [45].

**Figure 2:**
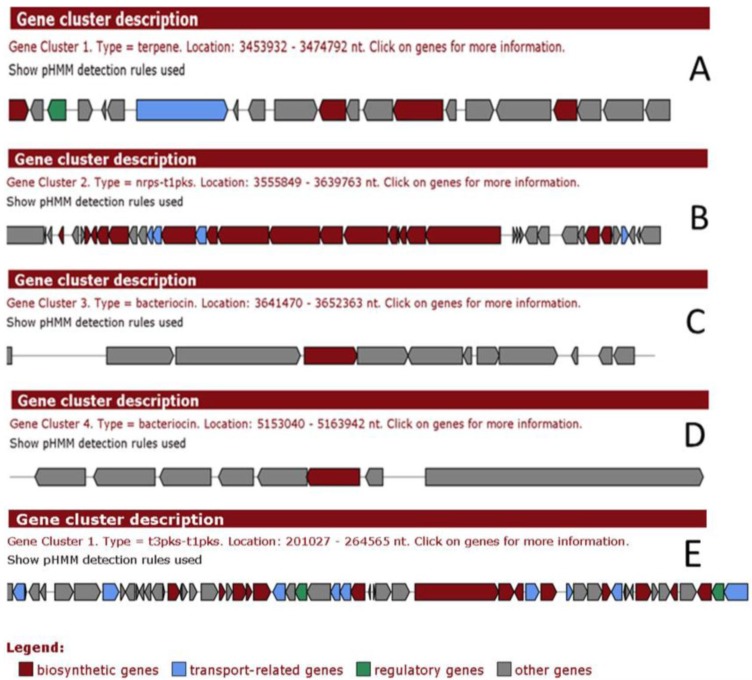
Analysis of *Pseudovibrio* FO-BEG1 via antiSmash showing (**A**) Terpene type gene cluster; (**B**) Non-ribosomal peptide synthetases (NRPS)-polyketide synthases (PKS) cluster; (**C**) Putative bacteriocin 1; (**D**) Putative bacteriocin 2 ; and (**E**) T3pks-T1pks cluster on plasmid.

Genomic analysis of *Pseudovibrio* FO-BEG1 (Figure 2) and JE062 has also revealed the potential for a diverse array of metabolic abilities within both strains [43].

Analysis revealed the presence of genes predicted to be involved in carbohydrate, lipid, fatty acid, lipopolysaccharide, sugar and glycan metabolism. Pathways involved in terpenoid, sterol, cofactor and vitamin and polyamine biosynthesis have also been identified, as have genes predicted to be involved in the biosynthesis of secondary metabolites such as monolignol, flavanone, flavonoid and paspaline [46]. Our own unpublished analysis [47], of *Pseudovibrio* FO-BEG1 *via* antiSmash (antibiotics and Secondary Metabolite Analysis Shell) and BAGEL (prediction of bacteriocins in prokaryotes) have highlighted a number of clusters of interest. More specifically, antiSmash highlighted a terpene type gene cluster (Figure 2A) and two putative bacteriocin clusters (Figure 2C,D), in addition to the previously mentioned *NRPS-PKS* system (Figure 2B) on the chromosome and a type T3pks-T1pks cluster on the plasmid (Figure 2E) [47]. BAGEL also highlighted loci of significance related to putative bacteriocins [48].

**Figure 3:**
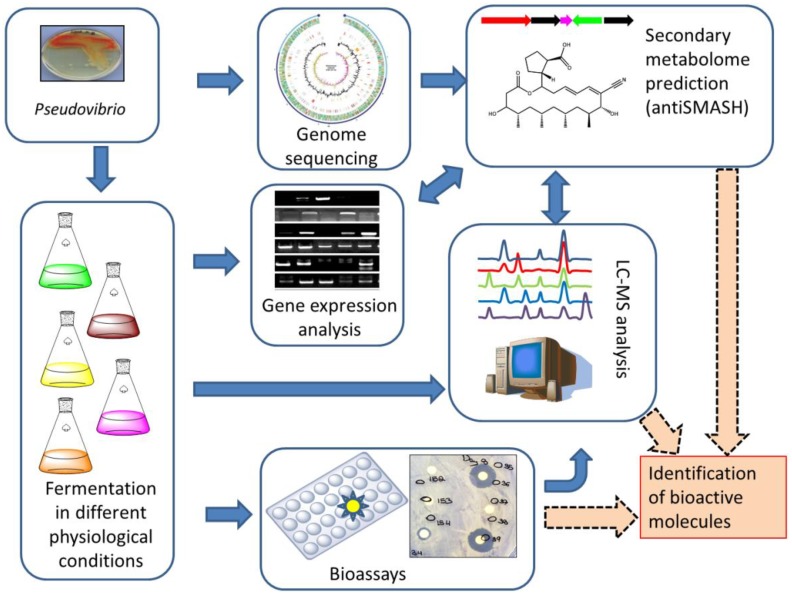
Schematic diagram outlining relevant experimental procedures which may lead to the identification of novel bioactive molecules.

Ultimately, although it is evident from the literature that *Pseudovibrio* sp. can possess broad range antimicrobial activity, in many cases the basis for this activity has not been further analysed. Therefore the opportunity to identify novel bioactive compounds exists. As previously mentioned, culture conditions can have a significant effect on the production of antimicrobials, which is often tightly regulated, and must be taken into consideration when designing experiments (Figure 3) in order to establish the optimum conditions for maximum production of bioactives. The schematic diagram below outlines the procedures currently underway and being employed to study a number of *Pseudovibrio* strains and which may prove fruitful for others.

In addition to the identification of potentially novel bioactive compounds, the use of these approaches can allow one to demonstrate the spectrum of antimicrobial activity of the strains, facilitate the identification of putative bioactive clusters and to carry out comparative genomics.

## 3. Conclusions

Antibiotic resistance among pathogenic microorganisms is becoming ever more common. Unfortunately, the development of new antibiotics which may combat these pathogens has decreased. Natural sources has provided, and can continue to provide, a diverse range of compounds for drug development and many of these biologically active natural products are of microbial origin. As the hunt for novel, natural and potent antibiotics continues, focus has recently shifted to the oceans for natural product discovery as the marine environment has revealed itself as the relatively untapped source of potent bioactives. In recent years, the marine-derived *Pseudovibrio* has been the focus of particular attention due to the dominance of the genus among host-associated microbial communities and its associated antimicrobial producing capabilities. More specifically, the potency, broad-spectrum and novelty of the compounds produced by these bacteria has highlighted this genus as a source of natural antimicrobial compounds which can potentially be employed to combat the prevalence of antibiotic resistant bacteria among the healthcare and food production sectors. Varying physiological growth conditions, gene expression and biological analysis, in addition to the knowledge gained through genome sequencing of members of the genus *Pseudovibrio*, may lead to the identification of genes involved in the production of secondary metabolites, optimum growth parameters and gene expression; eventually leading to the development of novel bioactives.
